# Abandonment of Post in Face of Enemy: Epilepsy

**Published:** 1916-10

**Authors:** 


**Abandonment of Post in Face of Enemy: Epilepsy.** Henri Verger, Jour. de Med. de Bordeaux, reports a case, with well marked personal and family history of epilepsy. The soldier had no recollection of his doings and returned to his station in the morning, when he was reproached by his companions. The author discusses the difficulties of diagnosis, especially in the brief time allowed for court martials.

